# An unusual case of an intramuscular lipoma of the biceps brachii

**DOI:** 10.11604/pamj.2013.15.40.2654

**Published:** 2013-06-02

**Authors:** Kamal Lahrach, Khalid Ibn el Kadi, Amine Mezzani, Amine Marzouki, Fawzi Boutayeb

**Affiliations:** 1 Department of Orthopedic Surgery (A), UH Hassan II, Fes, Morocco

**Keywords:** Intramuscular, lipoma, biceps brachii

## Abstract

Lipomas are common benign neoplasms consisting of mature fatty tissue. They are usually of roundish or ovoid shape and are situated in a single anatomical region. They most frequently occur on the back and in the extremities. Most lipomas are subcutaneous and require no imaging evaluation. When deep, large and unusual in location, MRI can identify and localise these tumours and is the best exploration to differentiate lipoma and lipo-sarcoma. We describe a case of a patient with an intramuscular lipoma of the biceps brachii.

## Introduction

Lipomas are the most frequent benign mesenchymal neoplasms with an estimated incidence of about 16% [1–3]. Intramuscular lipomas of the biceps brachii are rare tumors. The authors report one case and make a review of the diagnosis of this localization.

## Patient and observation

**Figure 1:**
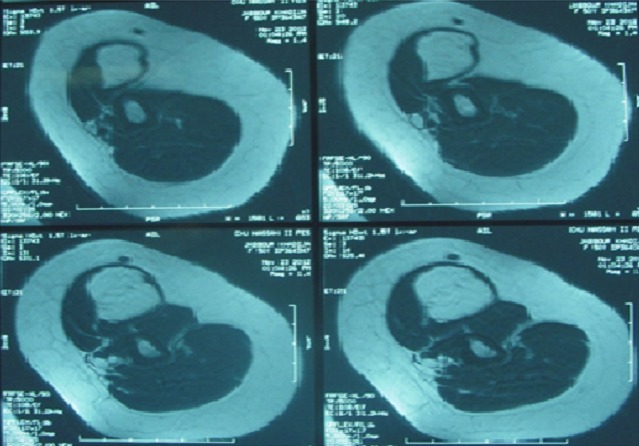
Preoperative magnetic resonance imaging showing an intramuscular lipoma of the biceps brachii muscle

**Figure 2:**
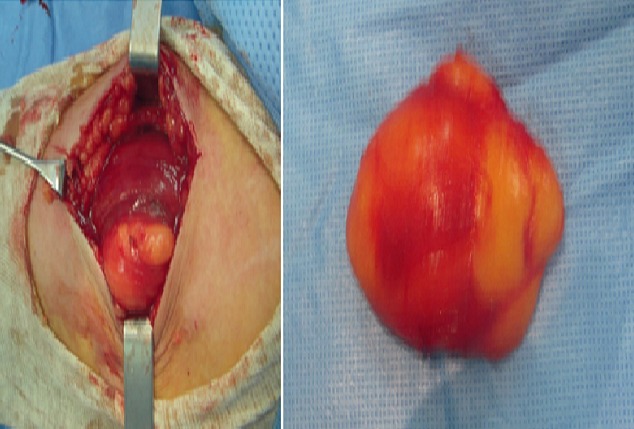
Intraoperative images of the excision. Final pathology revealed a be 8 cm × 6 cm × 4 cm lipoma

L.A. 50-years presented at our institution with a painless mass in her left arm region that she had noticed about six months earlier and that caused no other symptoms. The mass was nontender and he recalled no antecedent trauma. On physical examination, a deep, well-circumscribed mass was palpated in the region of the left biceps. The mass was freely movable, with no overlying skin changes. MRI revealed a mass within the left biceps brachii consistent with a low-grade lipomatous lesion, likely an intramuscular lipoma (Figure 1). The patient underwent radical excision of the lesion, which was found to be 8 cm × 6 cm × 4 cm in size (Figure 2). Final pathology revealed lipoma.

## Discussion

Lipomas are one of the most frequently encountered benign mesenchymal tumours composed of mature fat tissue, commonly found in the superficial subcutaneous tissues of the extremities and trunk [4]. Lipomas occur more frequently in female patients, presumably due to their tendency to accumulate more adipose tissue. Lipomas are slow-growing soft tissue tumours that rarely reach a size larger than 2 cm. Lesions larger than 5 cm, so-called giant lipomas, can occur anywhere in the body but are seldom found in the upper extremities [5]. A lipoma may occasionally be found within the muscle (intramuscular or infiltrating lipoma) or between the muscles (intermuscular lipoma). Intramuscular lipomas are extremely rare[6]. Intramuscular lipomas were first described in 1946 [7]. In most reported cases the tumours involved the extremities and trunk, or rarely involved different muscles in the head and neck region. Report on an intramuscular lipoma involving biceps brachiallii muscle could not be found in the literature. The exact mechanism for the increased growth of such lipomas is still in debate [8]. Most authors propose microtrauma that causes rupture of the fibrous septa and anchorage connections between the skin and deep fascia thus allowing the adipose tissue to proliferate [9]. Other theories, including the role of endocrine, dysmetabolic and genetic factors provoking the uncontrolled growth of giant cell lipomas, have been also proposed [10]. On MRI the lipoma appears as a non-invasive mass with homogenous fat signal intensity enveloped by a pseudocapsule [11]. Ultrasound investigations could also been used in detection of lipomas [8]. In cases of malignancy, MRI could distinguish a lipoma from a well-differentiated liposarcoma because of the increased levels of vascularity seen in septal structures within the malignant lesion [11]. The proper management of lipomas is open excision. Lipomas are usually well encapsulated, allowing relatively straightforward complete removal. Intramuscular location makes removal more technically challenging and may require removal of some surrounding muscle to ensure adequate margins. Finally, a full pathology report on the specimen is required to determine the possible need for further treatment [11–13].

## Conclusion

Intramuscular lipomas of the biceps brachii muscle are uncommon tumours. MRI can identify and localise these tumours. The treatment of lipomas consists of complete sur-gical removal.
